# Binary salp swarm algorithm for discounted {0-1} knapsack problem

**DOI:** 10.1371/journal.pone.0266537

**Published:** 2022-04-07

**Authors:** Binh Thanh Dang, Tung Khac Truong

**Affiliations:** 1 School of Engineering and Computer Science, Victoria University of Wellington, Kelburn, Wellington, New Zealand; 2 Faculty of Information Technology, Van Lang University, Ho Chi Minh City, Vietnam; Torrens University Australia, AUSTRALIA

## Abstract

While the classical knapsack problem has been the object to be solved by optimization algorithm proposals for many years, another version of this problem, discounted {0-1} knapsack problem, is gaining a lot of attention recently. The original knapsack problem requires selecting specific items from an item set to maximize the total benefit while ensuring that the total weight does not exceed the knapsack capacity. Meanwhile, discounted {0-1} knapsack problem has more stringent requirements in which items are divided into groups, and only up to one item from a particular group can be selected. This constraint, which does not exist in the original knapsack problem, makes discounted {0-1} knapsack problem even more challenging. In this paper, we propose a new algorithm based on salp swarm algorithm in the form of four different variants to resolve the discounted {0-1} knapsack problem. In addition, we also make use of an effective data modeling mechanism and a greedy repair operator that helps overcome local optima when finding the global optimal solution. Experimental and statistical results show that our algorithm is superior to currently available algorithms in terms of solution quality, convergence, and other statistical criteria.

## Introduction

Mathematical problems are not merely theoretical abstractions. Many of them are the mappings of real-life problems, and some have broad and practical applications. Hence, solving those problems becomes more urgent, and we can recognize the efforts of researchers in this area for many decades. Knapsack problem (KP) [1] is a typical example of such problems. In particular, KP is a classical combinatorial optimization problem in which we have a given set of items, and each item is coupled with a weight and a profit value.

The knapsack problem has vast practical applications in many areas. These applications include but are not limited to computer memory management, facilities management, energy consumption optimization, adaptive multimedia systems, resource allocation, logistics, encryption, cryptography, etc. All these seemingly unrelated problems meet in a common point: there is a limited resource and the utilization of that resource needs to be optimized.

To resolve the KP, we have to determine an item subset with the maximum sum profit while the total weight of the selected items is still less than or equal to a predetermined knapsack capacity. In Guldan’s thesis [2], new mathematical models of the discounted {0-1} knapsack problem (DKP01) are introduced. This problem integrates the discount concept used in the real-world sale business, in which we can get a discounted price for purchasing multiple items together.

In DKP01, assume that we have *n* groups with three items each. Each item has two features. These features were often referred to as profit and weight in previous works. Although these names are consistent with the idea of the capacity of a knapsack in the original KP, they do not really fit the cost reduction concept in the DKP01. Therefore, we suggest that this pair of features be value and cost, abbreviated as *v* and *c*. Items in group *i*, *i* ∈ {0, 1, …, *n* − 1}, can be modeled as *x*_*i*,1_(*c*_*i*,1_, *v*_*i*,1_), *x*_*i*,2_(*c*_*i*,2_, *v*_*i*,2_), and *x*_*i*,3_(*c*_*i*,3_, *v*_*i*,3_). Note that the third item, *x*_*i*,3_, represents the case where the discount is applied in real life, with *c*_*i*,1_ + *c*_*i*,2_ > *c*_*i*,3_ and *v*_*i*,3_ = *v*_*i*,1_ + *v*_*i*,2_. In other words, the situation where item *x*_*i*,3_ is chosen is equivalent with the case where both of the first and the second item in the group, *x*_*i*,1_ and *x*_*i*,2_, are selected and, for choosing them together, we get a discounted cost. As a result, for each group, one in four options can happen: (i) taking the first item only, (ii) taking the second item only, (iii) taking the third item (which equals to taking both the first and second item in real world) and get the discounted cost *c*_*i*,3_, and (iv) not taking any in these items at all. In another way, in the presentation model of the problem where there are three items in a group, we can select at most one item from that group. This is what separates DKP01 from the traditional KP, which does not divide items into groups and does not have any limitation in terms of the number of items to be selected in each group. This particular characteristic makes DKP01 more strenuous than the original KP.

Generally, the goal in solving DKP01 is selecting a subset of items whose total value is maximum while the sum cost of the chosen ones is not greater than a predefined threshold *C* and no more than one item can be picked from each group. This problem is mathematically modeled as follows.

$$\begin{array}{c} Maximize\;f\left(X\right)=\sum_{i=0}^{n-1}\left(s_{i,1}v_{i,1}+s_{i,2}v_{i,2}+s_{i,3}v_{i,3}\right) \end{array}$$ (1)

$$\begin{array}{c} Subject\;to\;s_{i,1}+s_{i,2}+s_{i,3}\leq 1,\;i\in \left\{0,1,..,n-1\right\} \end{array}$$ (2)

$$\begin{array}{c} \sum_{i=0}^{n-1}\left(s_{i,1}c_{i,1}+s_{i,2}c_{i,2}+s_{i,3}c_{i,3}\right)\leq C \end{array}$$ (3)

$$\begin{array}{c} \;s_{i,1},s_{i,2},s_{i,3}\in \left\{0,1\right\},\forall i\in \left\{0,1,\ldots ,n-1\right\} \end{array}$$ (4)

where, *s*_*i*,*j*_ = 0 shows that the item *x*_*i*,*j*_ is not chosen, and *s*_*i*,*j*_ = 1 means the item *x*_*i*,*j*_ is selected, with *i* ∈ {0, 1, …, *n* − 1}, *j* ∈ {1, 2, 3}. A binary vector *A* = (*a*_0_, *a*_1_, …, *a*_3*n*−1_) ∈ {0, 1}^3*n*^ is a candidate solution of DKP01 if *A* complies with the conditions given by Eqs 2 and 3.

Being an NP-hard problem, unless P = NP, DKP01 cannot be resolved using a polynomial-time algorithm. In her master’s thesis, Guldan proposes the DKP01 and uses dynamic programming to solve it [2]. in fact, the set-out cases of the KP have very different properties. Circumstances where items have a weak relationship, or even no relationship, between value and cost, are considered relatively easy to find the optimal solution even when the number of items is large. Meanwhile, in cases where values and costs are closely related, choosing which items to put in the knapsack is harder. Based on this characteristic, one way to solve the traditional KP is using the idea of core, which is defined in [3, 4]. Then, the authors of [5] define an alternative core of the DKP01 by mimicking the idea of core in KP. Using this new alternative core, they propose a partitioning scheme to divide the original DKP01 into sub-problems to reduce calculation complexity, and utilize dynamic programming to solve them. Additionally, [6] proposes an exact algorithm that tries to minimize the total cost with a predetermined sum value to solve DKP01. Then, based on this algorithm, three approximate algorithms are introduced.

Evolutionary and swarm intelligence-based computation are also applied in solving DKP01. The authors of [7] propose two mathematical models for DKP01 and two genetic algorithm-based algorithms, FirEGA and SecEGA, to resolve the problem. In [8], the authors propose two evolutionary operators called global exploration operator (R-GEO) and local development operator (R-LDO) to design a ring theory-based evolutionary algorithm which is used to solve the DKP01. While the two operators rely on ring theory, the evolutionary algorithm is based on the flower pollination algorithm [9]. The authors of [10] also nominate a multi-strategy algorithm for DKP01 on the basis of monarch butterfly optimization (MBO) [11]. In this study, the monarch butterfly population was separated into two sub-populations. The positions of monarch butterfly individuals in the first sub-population are handled by a neighborhood mutation-based crowding operator, which replaces the original MBO migration operator. Moreover, in [12], the application of moth search (MS) for DKP01 is investigated. First, the impacts of the Lévy flights operator and the fly straightly operator on basic MS are evaluated. Then, nine MS-based algorithms are developed using a global-best harmony search (GHS)-based mutation operator. Another contribution in nature-inspired optimization algorithm application, [13], introduces a discrete hybrid teaching-learning-based optimization algorithm (HTLBO) to resolve DKP01. A quaternary code is introduced to represent a DKP01 solution, and the individuals are modeled by double coding. The Learner’s learning strategy is improved to expand the discovery capabilities of HTLBO, while self-learning is implemented to balance exploration and exploitation. Two sorts of crossover are also designed to strengthen the effectiveness of global search in this algorithm.

In a recently published work [14], Truong has developed a binary version of the famous Particle Swarm Optimization algorithm [15] to solve the DKP01 problem. In another publication [16], moth-flame optimization [17] is used to solve this problem. Most recently, an improved version of the Harris Hawks Optimization (HHO) algorithm [18] is proposed in [19]. Although the HHO algorithm has a pretty good balance between exploration and exploitation, the authors of this paper have suggested tweaks that target the attack phase of Harris hawks using opposition-based learning (OBL) strategy to increase the diversity in the search process. The main idea of OBL is to compare the fitness values of the current solution and its opposite case and then choose the better solution to include in the next generation. Additionally, the prey escape energy value, which was originally designed to reduce linearly, has also been redefined to reduce logarithmically non-linearly, making the transition between exploration and exploitation smoother. The authors also introduce a random unscented sigma point mutation mechanism to help HHO converge more quickly to the best solution the algorithm can achieve. Besides solving traditional benchmark functions (CEC2017 and CEC2020) and engineering problems, the resulting algorithm is also used to solve the DKP01 problem in selected data sets. However, the test results show that the existing DKP01 test instances are not simple, and this algorithm has not achieved very good results, which also means that there is still a lot of space for other solutions in the future.

Besides taking advantage of classical optimization algorithms, new optimization algorithms are also regularly introduced and open up new directions in solving optimization problems. An example for this is [20], where the authors presented two variants of a widely accepted swarm intelligence-based optimization algorithm, the single objective salp swarm algorithm (SSA) and the multi-objective salp swarm algorithm (MSSA). The main motivation of SSSA and MSSA is the swarming conduct of salps when exploring and rummaging for food in the seas. Test results on various data sets show that the SSA is able to improve the initial arbitrary solutions and converge towards the ideal one. More details on SSA will be given in the next section of this paper.

Despite being a relatively new algorithm, SSA has been cited in several scientific works across various research fields. In [21], the authors develop a binary version of SSA utilizing eight transformation functions and a crossover operator instead of the basic one which the original SSA provides. In [22], to study the optimal connections between switches and controllers and the optimal number of deployed controllers in large-scale software-defined networks (SDN) [23], the authors propose an optimization algorithm based on SSA using chaotic maps. In an effort to solve the feature selection problem, [24] introduces another chaotic SSA algorithm and integrates it with a K-nearest neighbor classifier. Their solution is also proved to be efficient in tackling the local optima stagnation issue as well as improving the convergence behavior of the original SSA algorithm. [25] implements opposition-based learning in the initialization phase of SSA to enhance its population diversity. Moreover, local search algorithm (LSA) is also used in this work to improve exploitation performance. The authors of [26] propose a binary SSA using a modified arctan transformation. In [27], SSA is enhanced by balancing the exploration and exploitation process. [28] extends the original SSA by implementing multiple independent salp chains and applies them for maximum power point tracking (MPPT) of photovoltaic systems under partial shading conditions. [29] uses space transformation search (STS) [30] to improve the performance of SSA, and the resulted algorithm is deployed to train a multi-layer feed-forward network. A recent publication, [31], proposes new mutation operators to balance the exploration and exploitation phases of SSA. The authors of [32] present the solitary and colonial reproduction phase of salp in emended salp swarm algorithm (ESSA), which is used to resolve the economic load dispatch problem in a multi-objective framework. In [33], composite mutation strategy (CMS) and restart strategy (RS) are integrated into SSA to boost exploitation and exploration trends of SSA as well as aid salps in avoiding local optimum.

Though numerous studies have referred to SSA, to the best of our knowledge, this paper is the first to utilize SSA in resolving DKP01. Although the algorithms for DKP01 mentioned above have achieved encouraging results, parts of the solutions chosen by them are not very reasonable. They can be improved, such as the classical solution representation, which is not an ideal choice and will be replaced by the scheme in this paper. Besides, we intend to combine the power of SSA with the application of a greedy repair operator for local optimization as well as to address the weakness of SSA when its solutions are easily stuck at the local optimal point and can’t get out to try other candidate solutions during the global optimization process. In detail, the contributions of this work include:

- A novel binary salp swarm algorithm (BSSA) with four binary transformation functions and a new solution presentation scheme to solve the discounted {0-1} knapsack problem.
- A combination with a minimal encoding scheme whose binary solution vector length is 2*n* (in comparison to the length of 3*n* of the original DKP01 that is used in many previous papers). While providing enhancements in calculation speed and reducing the complexity, this scheme automatically satisfies the constraint of the DKP01 stated in Eq 2.
- The use of a repair operator on the positions of the salps during salp chain movement towards the food source to avoid local optima and enhance calculation effectiveness.

The rest of this paper is as follows. The next section gives an introduction to the salp swarm algorithm (SSA), which is the basis for our algorithm. The section after that details our proposed binary SSA for DKP01. Then come the simulation results and discussion of our algorithm’s performance in comparison to those of other existing algorithms. Finally, the Conclusions section will conclude the paper.

## Salp swarm algorithm

Introduced by [20], SSA has received much attention recently due to its simplicity, effectiveness, as well as adaptability to various optimization problems. This section will give details on this algorithm.

A salp, which can be found generally in deep seas but sometimes near the surface, is a barrel-formed, planktic tunicate that moves by contracting, thereby pushing water through its jelly-like body. One of the most interesting activities of a salp population is forming a salp chain, which may increase the swarm effectiveness in traveling and foraging. SSA is an effort to facsimile the swarming behavior of salps in oceans.

In SSA, individuals in a salp population are classified into two categories: the leader, which is the salp at the head of the chain, and the followers. The position of a given salp is modeled as an *n*-dimensional search space, in which *n* is the number of variables of the problem to be solved. As a result, all the position vectors of the salp population form a 2-dimensional matrix named *pos*. The food source of the swarm is modeled as the target *F* in the search space.

The position of the leader is updated utilizing the below condition:

$$\begin{array}{c} pos_{j}^{1}=\{\begin{array}{cc} F_{j}+c_{1}\left(\left(ub_{j}-lb_{j}\right)c_{2}+lb_{j}\right), & c_{3}\geq 0.5\\ F_{j}-c_{1}\left(\left(ub_{j}-lb_{j}\right)c_{2}+lb_{j}\right), & c_{3}<0.5 \end{array} \end{array}$$ (5)

where $pos_{j}^{1}$ represents the coordinates of the leader in the *j*th dimension, *F*_*j*_ depicts the position of the target *F* in the *j*th dimension, *ub*_*j*_ is the upper bound of the *j*th dimension, and *lb*_*j*_ is the lower bound of the *j*th dimension. Additionally, *c*_1_ is a number generated using the following rule:

$$\begin{array}{c} c_{1}=2e^{-{\left(\frac{4k}{K}\right)}^{2}} \end{array}$$ (6)

where *k* is the current iteration and *K* is the maximum iteration. Meanwhile, *c*_2_ and *c*_3_ are randomized in the range [0, 1].

The positions of the followers are manipulated using the below equation:

$$\begin{array}{c} pos_{j}^{i}=\frac{1}{2}(pos_{j}^{i}+pos_{j}^{i-1}) \end{array}$$ (7)

The general idea of SSA is simple: the leader moves towards the target (food source), and the followers trail the leader. In optimization problems, while the global optimum should be the target, there is no such thing that exists. To resolve this, the best solution obtained at a given time is considered the global optimum, and the salps should head towards it. The pseudo-code of SSA is shown in Algorithm 1.

**Algorithm 1**: The pseudo-code of salp swarm algorithm

**Inputs**: Initial parameters

**Output**: Optimal solution

Initialize salp population considering upper bound and lower bound

**while** *end condition is not met* **do**

 F ← the best search agent (salp)

 Generate *c*_1_ using Eq 6

 **for** *each salp* **do**

  **if** *the salp is the leader* **then**

   Update the position of the leading salp by Eq 5

  **else**

   Update the position of the current salp by Eq 7

  Amend the salps based on the upper and lower bounds of variables

**return** *F*

Next, we will do some analysis to clarify how SSA works. From the given information, the interesting part is how the position elements of the salps are manipulated. Firstly, it is easy to notice that the lower bound *lb* and upper bound *ub* vectors are critical in keeping the position elements of the leading salp be in the valid range, which will, in turn, lead the followers on the right path. For simplicity, assume that all items in *lb* has the same value of 1, and all items in *ub* has the same value of 10. Thus, from Eq 5 and since *c*_2_ is randomly generated in [0, 1], the position of the leader in the *j*th dimension is specified by:

$$\begin{array}{c} pos_{j}^{1}=\{\begin{array}{c} p_{1},p_{1}\in \left[F_{j}+c_{1},F_{j}+10c_{1}\right],\;\text{with}\;\text{probability}\;\frac{1}{2}\\ p_{2},p_{2}\in \left[F_{j}-10c_{1},F_{j}-c_{1}\right],\;\text{otherwise} \end{array} \end{array}$$ (8)

**Fig 1:**
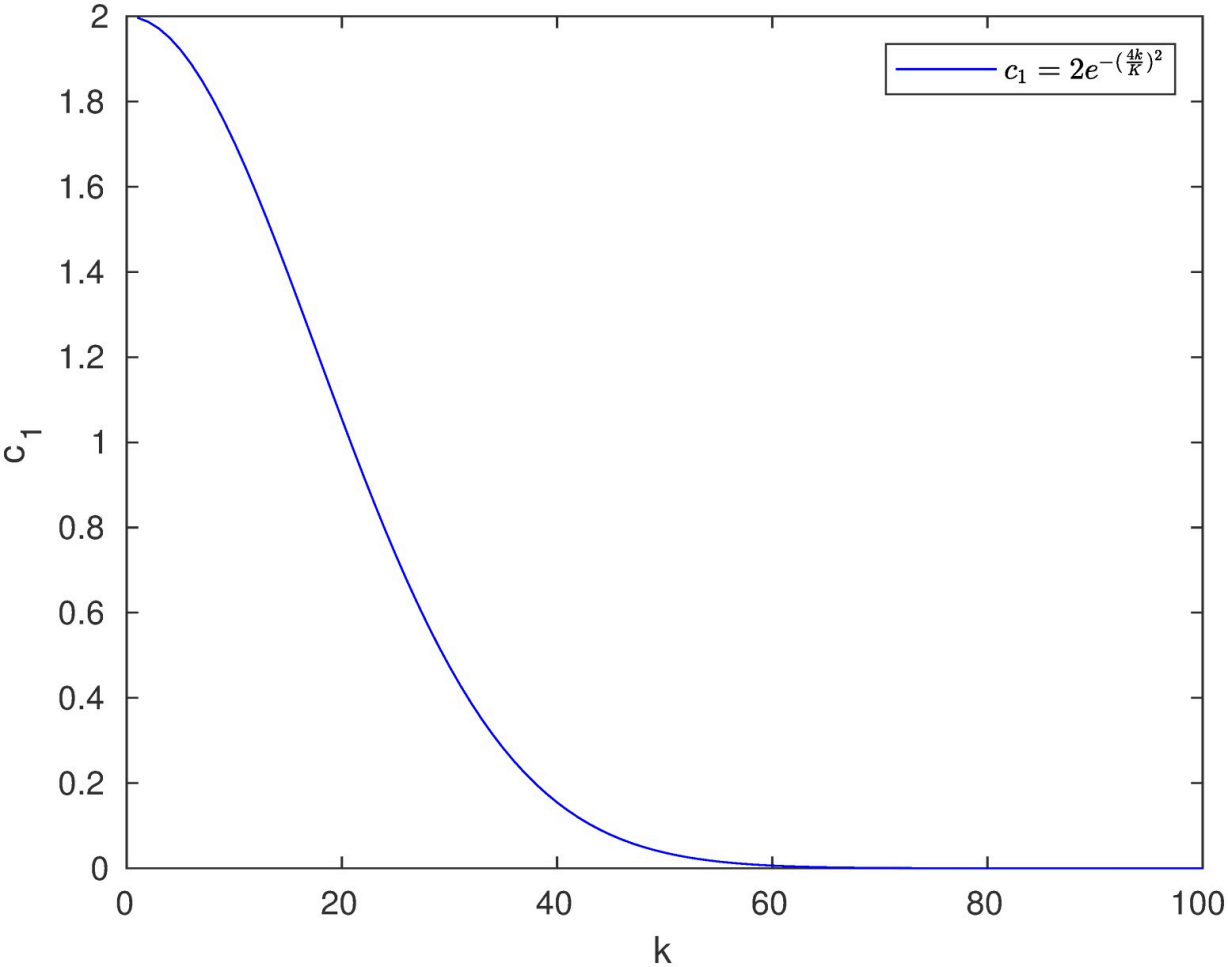
Change in value of *c*_1_ during iteration.

The values of *c*_1_ are in the range of [0, 2]. Assume that the maximum iteration *K* = 100, the curve formed by values of *c*_1_ is illustrated in Fig 1.

It can be seen that the closer *k* gets to *K*, the smaller *c*_1_ is, and the less chance that the corresponding position element of the leading salp can change significantly. Although this is consistent with the nature of global search in evolutionary computation, where searches in the early stage should cover a broader scope than those in the later stage, this also represents the risk that the leading salp can be easily stuck at a local optimal point. This becomes even more serious when a position element of a following salp is simply the average between the value of the new position element of the preceding salp and the value of its own position element in the previous iteration. This means that, when the leading salp gets stuck, it is unlikely that the salps that follow it have a way to assist it in coming up with solutions to get out of the local optimum.

To resolve this problem and due to the fact that SSA has no mechanism to deal with DKP01 constraints, we decide to implement a repair operator which will help the solution given by SSA avoid local optima, and improve its fitness. Details of this operator and other proposed algorithms are given in the next section. Note that although there exists a multi-objective version of SSA, discussion of it is beyond the scope of this paper.

## Proposed binary salp swarm algorithm for DKP01

The original SSA needs many amendments to solve the DKP01. This section will provide details on the solutions that we propose.

### Binary transformation functions

To operate in a binary search space, binary transformation functions are necessary so that the related parameters should take the value 0 or 1 only. Sigmoid function [34, 35] is widely accepted as a means of transferring real values into probability. In this paper, we utilize four S-shaped sigmoid transformation functions as follows:

$$\begin{array}{c} Sig_{1}\left(z\right)=\frac{1}{1+e^{-2z}} \end{array}$$ (9)

$$\begin{array}{c} Sig_{2}\left(z\right)=\frac{1}{1+e^{-z}} \end{array}$$ (10)

$$\begin{array}{c} Sig_{3}\left(z\right)=\frac{1}{1+e^{-\frac{1}{2}z}} \end{array}$$ (11)

$$\begin{array}{c} Sig_{4}\left(z\right)=\frac{1}{1+e^{-\frac{1}{3}z}} \end{array}$$ (12)

**Fig 2:**
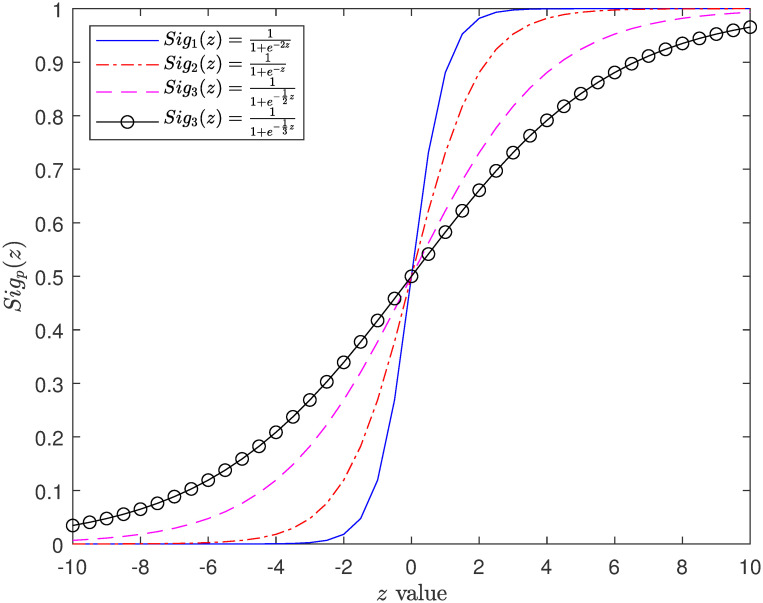
Curves of sigmoid functions used in our proposed algorithm.

Plots drawn from the outputs of functions detailed in Eqs 9–12 are shown in Fig 2. Using these four functions, we propose a novel Binary SSA (BSSA) optimizer for DKP01 which has four variants being BSSA1, BSSA2, BSSA3, and BSSA4, respectively. In particular, BSSA1 will take advantage of *Sig*_1_(⋅), BSSA2 utilizes *Sig*_2_(⋅), BSSA3 implements *Sig*_3_(⋅), and BSSA4 makes use of *Sig*_4_(⋅). Our new algorithm will also use modified versions of Eqs 5 and 7 which will use the transformation functions given in Eqs 9–12.

Firstly, we define *z*_1_ and *z*_2_ as:

$$\begin{array}{c} z_{1}=\{\begin{array}{cc} F_{j}+c_{1}\left(\left(ub_{j}-lb_{j}\right)c_{2}+lb_{j}\right), & \text{with}\;\text{probability}\frac{1}{2}\\ F_{j}-c_{1}\left(\left(ub_{j}-lb_{j}\right)c_{2}+lb_{j}\right), & \text{otherwise} \end{array} \end{array}$$ (13)

$$\begin{array}{c} z_{2}=\frac{1}{2}(pos_{j}^{i}+pos_{j}^{i-1}) \end{array}$$ (14)

As a result, we have new expressions for salp position manipulation as follows:

$$\begin{array}{c} pos_{j}^{1}=\{\begin{array}{cc} 0, & r\geq Sig_{p}\left(z_{1}\right)\\ 1, & r<Sig_{p}\left(z_{1}\right) \end{array} \end{array}$$ (15)

$$\begin{array}{c} pos_{j}^{i}=\{\begin{array}{cc} 0, & r\geq Sig_{p}\left(z_{2}\right)\\ 1, & r<Sig_{p}\left(z_{2}\right) \end{array} \end{array}$$ (16)

where *p* ∈ {1, 2, 3, 4}, and *r* is a randomized value, *r* ∈ [0, 1].

### Solution presentation

The traditional approach to encode a solution of a {0, 1} optimization problem is using a binary vector whose length is the number of dimensions of the search space:

$$\begin{array}{c} T=\left\{t_{1},t_{2},\ldots ,t_{3n}\right\}\in {\left\{0,1\right\}}^{3n} \end{array}$$ (17)

Each three-bit binary number represents three items in a group. If a bit is set to 1, the item at the related spot is selected. Otherwise, value 0 at a given bit means that the item is not chosen to be in the knapsack.

In this paper, we use a binary encoding scheme as shown in Table 1, with two-bit binary numbers used for solution presentation, as described in Eq 18.

$$\begin{array}{c} Y=\left\{y_{1},y_{2},\ldots ,y_{2n}\right\}\in {\left\{0,1\right\}}^{2n} \end{array}$$ (18)

**Table 1:** Binary encoding scheme.

No.	Binary value	Description
1	00	No item in the group is selected
2	01	The first item in the group is selected
3	10	The second item in the group is selected
4	11	The third item in the group is selected

For binary solutions of length 3*n*, the search space has 2^3*n*^ possible cases, while a binary solution of length 2*n* has a much smaller search space: 2^2*n*^ potential cases. The representation of the 2*n* solution allows the search for candidate solutions in a much smaller space. Besides, the representation of the 2*n* solution also helps them to automatically satisfy the constraint specified in Eq 2, which we do not have when using the 3*n* representation. 3*n* solutions need to be checked to ensure that a specific three-bit binary number does not violate Eq 2. In the worst case, when a violation occurs, another value needs to be assigned to that number. These actions are not necessary with binary solutions of length 2*n*.

When considering the constraint of Eq 2, all random solutions in the 2*n* search space are possible solutions, while the 3*n* search space contains non-viable solutions. Therefore, representing a solution of length 2*n* reduces the computation time.

### Repair operator

To deal with the restriction in Eq 3 and enhance the solution, we use a repair operator based on the functions used in [6, 14]. With *n* groups, we have a total of 3*n* candidate items to be put into the knapsack, including the combined items. Note that when the mentioned functions only support the 3*n* solution, we design our operator so that the 2*n* solution is supported while 3*n* items are still in consideration.

In short, the repair operator does the job of manipulating the selected set based on the value-to-cost ratio values *v*_*i*, *j*_/*c*_*i*, *j*_, (*i* ∈ {0, 1, …, *n* − 1}, *j* ∈ {1, 2, 3}) to reduce CPU usage and improve local optimum avoidance capability.

Since choosing which items to remove from or to add to the knapsack is not simply a matter of prioritizing combined items (a particular combined item is not necessarily better than a single item), we decide to sort all items and put them into a deterministic process. Thus, before the repair operator execution, all the items, including the combined ones, are sorted decreasing by the value-to-cost ratio values. The indexes of the items in this order are kept in the *ID* vector of length 3*n*. Using the *ID* vector, the items with more priority will be processed first. Then, the steps which this operator will do are as follows.

The repair operator has two phases: the repair and optimization phases. The repair phase is designed to fix a solution to become a feasible one from an impracticable state. Meanwhile, the optimization phase will enhance the fitness of a viable solution. If the current total cost is greater than *C*, the repair phase will remove items from the knapsack until the condition given by Eq 3 is met. After that, the optimization phase adds items to the knapsack provided that the total cost does not exceed *C*.

The inputs of the operator include the solution *Y* of length 2*n*, the cost vector of length 3*n*, the index vector *ID*, and the knapsack capacity *C*.

Algorithm 2 shows the pseudo-code of the related repair operator. Note that the operator’s computational complexity is *O*(*n*).

**Algorithm 2**: Repair operator

**Input**: Solution *Y* = (*y*_1_, *y*_2_, …, *y*_2*n*_) ∈ {0, 1}^2*n*^, cost vector *c*, index vector

   *ID* = (*id*_1_, *id*_2_, …, *id*_3*n*_), and knapsack capacity *C*.

**Output**: Solution after repair *Y*

% Repair phase

*f*_*c*_ ← 0

**for** *k* ← 1 *to* 3 * *n* **do**

 *i* ← *floor*((*id*_*k*_ − 1)/3)

 *j* ← *mod*(*id*_*k*_ − 1, 3) + 1

 **if** *y*_2*i*+1_ = 0 ∧ *y*_2*i*+2_ = 1 ∧ *j* = 1 **then**

  **if** $f_{c}+c_{id_{k}}\leq C$ **then**

$f_{c}\leftarrow f_{c}+c_{id_{k}}$

  **else**

   *y*_2*i*+1_ ← 0; *y*_2*i*+2_ ← 0

 **if** *y*_2*i*+1_ = 1 ∧ *y*_2*i*+2_ = 0 ∧ *j* = 2 **then**

  **if** $f_{c}+c_{id_{k}}\leq C$ **then**

$f_{c}\leftarrow f_{c}+c_{id_{k}}$

  **else**

   *y*_2*i*+1_ ← 0; *y*_2*i*+2_ ← 0

 **if** *y*_2*i*+1_ = 1 ∧ *y*_2*i*+2_ = 1 ∧ *j* = 3 **then**

  **if** $f_{c}+c_{id_{k}}\leq C$ **then**

$f_{c}\leftarrow f_{c}+c_{id_{k}}$

  **else**

   *y*_2*i*+1_ ← 0; *y*_2*i*+2_ ← 0

% Optimization phase

**for** *k* ← 1 to 3 * *n* **do**

 *i* ← *floor*((*id*_*k*_ − 1)/3)

 *j* ← *mod*(*id*_*k*_ − 1, 3) + 1

 **if** $y_{2i+1}=0\wedge y_{2i+2}=0\wedge f_{c}+c_{id_{k}}\leq C$ **then**

$f_{c}\leftarrow f_{c}+c_{id_{k}}$

  **if** *j* = 1 **then**

   *y*_2*i*+1_ ← 0; *y*_2*i*+2_ ← 1

  **if** *j* = 2 **then**

   *y*_2*i*+1_ ← 1; *y*_2*i*+2_ ← 0

  **if** *j* = 3 **then**

   *y*_2*i*+1_ ← 1; *y*_2*i*+2_ ← 1

**return** *Y*

To sum up, the pseudo-code of our proposed BSSA algorithms for DKP01 is detailed in Algorithm 3.

**Algorithm 3**: Pseudo-code for BSSA algorithms for DKP01

**Input**: Initial parameters

**Output**: Optimal solution

Initialize salp population considering upper bound and lower bound

**while** *end condition is not met* **do**

 **for** *each salp* **do**

  Converting real position values of the current salp into binary numbers using Eqs 15 or 16

  Calculate the fitness of the current salp using the repair operator

 F ← the best salp

Generate *c*_1_ using Eq 6

**for** *each salp* **do**

  **if** *the salp is the leader* **then**

   Update the position of the leading salp by Eq 5

  **else**

   Update the position of the current salp by Eq 7

Amend the salps based on the upper and lower bounds of variables

## Results and discussion

The simulations used for this paper are for these goals:

- Compare four variants of our proposed BSSA to determine the best ones for DKP01. This is an internal test only. Thus, only the algorithms proposed by this paper are included in related tests, diagrams, and tables.
- Then, our best BSSA variants for DKP01 will be compared to selected algorithms proposed by other scientific works to see which one performs best in various aspects through statistical calculations. The chosen algorithms are the best we could find in recent publications.

Firstly, we choose two revised versions of genetic algorithm (GA) [36] and particle swarm optimization (PSO) [15] to include in the comparison. In the case of the GA variant for DKP01, we choose FirEGA, which is introduced in [7]. The PSO version to be tested is the best one from [14], BPSO8. We also include the results of MS1, which is designed based on the moth search algorithm and is the best algorithm for DKP01 proposed in [12], and MMBO, a multi-strategy monarch butterfly optimization algorithm for DKP01 introduced by [10]. The authors of this paper propose many variants of their algorithm, and we choose the best one of them. In [19], the authors have tested their algorithm on selected instances of the DKP01 problem. Since this is a promising algorithm and a recently published work, we also decided to include the experimental results of this algorithm for comparison.

The parameters used for testing are shown in Table 2. For a fair comparison, we set the population sizes (the number of particles in case of the PSO variant) at the same value, 50. Furthermore, the maximum iterations of all algorithms are set to the number of dimensions of DKP01, 2*n*, for the same reason.

**Table 2:** Simulation parameters.

Algorithm	Parameter	Variable Name	Value
FirEGA	Probability of crossover	*p*_*c*_	0.8
	Probability of mutation	*p*_*m*_	0.01
BPSO8	Learning factors	*c*_1_ and *c*_2_	2
	Inertia weight	*w*	0.9,…,0.4
IHHO	Population size	*N*	50
MS1	Maximum step	*S*_*max*_	1
	Acceleration factor	*φ*	0.618
	Index	*β*	1.5
MMBO	Neighborhood size	*m*	5
	Migration ratio	*p*	5/12
	Butterfly adjusting rate	*BAR*	5/12
	Maximum step	*S*_*max*_	1
BSSA	Lower bound	*lb*	1
	Upper bound	*ub*	10

We use 40 DKP01 instances proposed by [7] and available at https://www.doi.org/10.6084/m9.figshare.19416857.v2 to test all algorithms. They include 10 strongly correlated instances (SDKP1-SDKP10), 10 inverse strongly correlated instances (IDKP1- IDKP10), 10 uncorrelated instances (UDKP1- UDKP10), and 10 weakly correlated instances (WDKP1- WDKP10). The correlation is considered strong when cost and value are closely related and highly dependent on each other. Contrarily, the correlation is considered weak when cost and value are loosely related. The number of items in each instance is 3*n*, *n* ∈ {100, 200, …, 1000}. The mentioned instances are also used in [37].

All related algorithms, coded on MATLAB R2018a, run on an ASUS laptop, equipped with an Intel Core i5-8250u 1.6 GHz CPU, 8 GB DDR3 SDRAM, and uses Microsoft Windows 10 as the operating system.

### Convergence behaviour

Our first concern is the convergence speed towards the optimal solution of the algorithms. We recorded the degree of convergence by running four versions of our proposed algorithm on different data set files, each algorithm being run once. After each iteration, the resulting best-so-far total value is saved. This set of values is fed into a graph showing the convergence behaviour.

**Fig 3:**
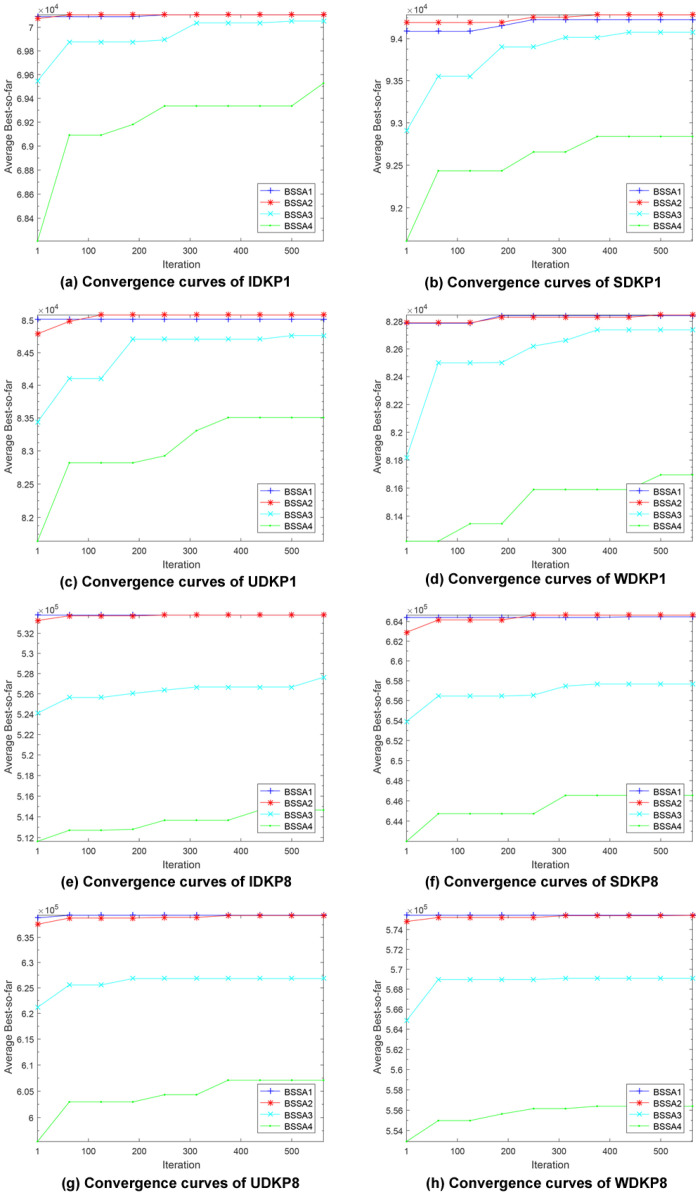
Convergence curves.

In fact, the four data set types of the DKP01 problem have quite different characteristics. However, we found that the convergence behavior of these algorithms on problems of different sizes on the same data set type is not significantly different. Therefore, we decided to choose two typical cases to describe the convergence of the algorithms for each type of data set. Fig 3 summarizes the converging curves for these types of data sets.

In the test with all data sets, BSSA1 and BSSA2 proved their superiority over the other two versions of the algorithm. They achieve better solution quality and higher fitness value from the first iterations. The early convergence behaviour also suggests that BSSA1 and BSSA2 can be further improved to take advantage of later iterations. Fig 3 also shows that while BSSA3 and BSSA4 can perform closer to the performances of BSSA1 and BSSA2 in case of smaller problems, it can be concluded that BSSA3 and BSSA4 are not appropriate to be used for larger problems.

### Stability and solution quality

This subsection focuses on examining the stability and quality of the solutions returned by our proposed algorithms. For demonstration, we use box plots whose data are the best values achieved after each algorithm run. To obtain a series of best values that will be used to create the box plot, we run each algorithm 30 times and get 30 best results.

In descriptive statistics, a box plot [38] is a graphical tool to demonstrate the data distribution using a five-number summary of that data set. Those five numbers are the minimum, the first quartile, the median, the third quartile, and the maximum values. A box plot will occupy the space from the first quartile to the third quartile, and as a result, it will span approximately 50 percent of the data range from the minimum value to the maximum one. The lowest 25 percent and the highest 25 percent spaces are not in the box. The horizontal line in the box stands for the median. The higher this line is, the better the quality will be. Moreover, the more flattened the box, the more consistent the values.

**Fig 4:**
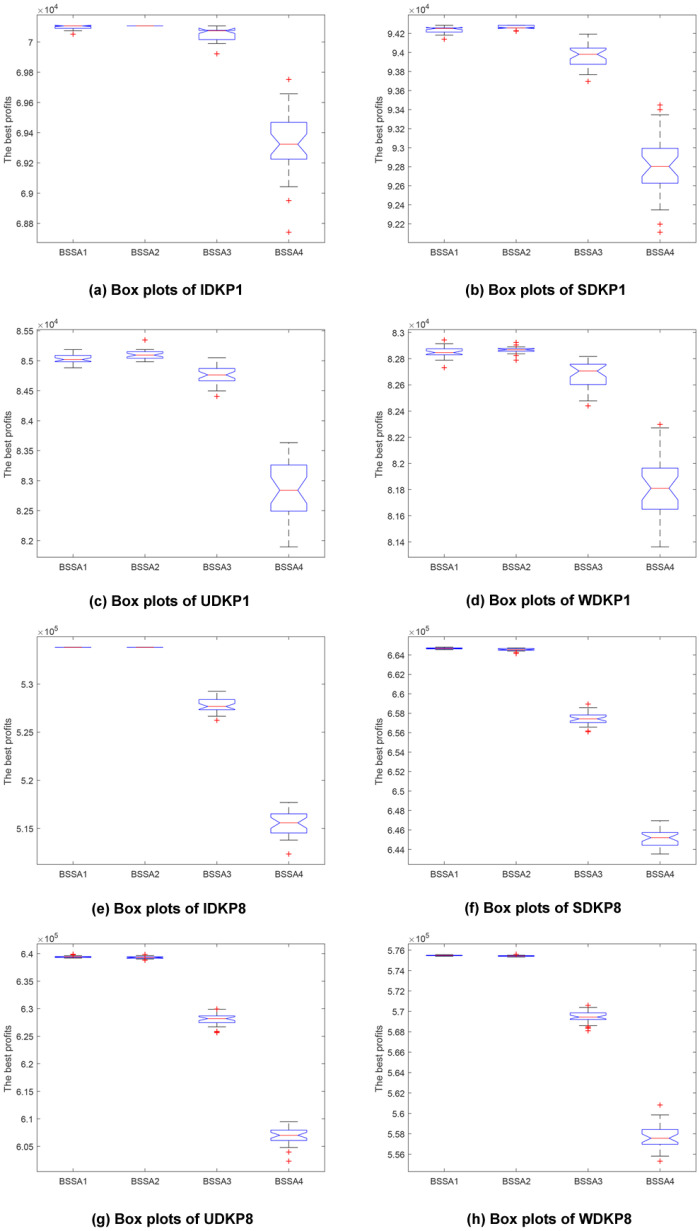
Box plots.

We use the same approach as in the analysis of the convergence curves, which means that we choose two typical cases for each data set type. Fig 4 shows these charts. It is easy to see that BSSA3 and BSSA4 cannot compete with BSSA1 and BSSA2. Their boxes are thicker, which means the outputs are not stable. In other words, the differences among best total values obtained after 30 runs of these algorithms are significant. In most cases, even the maximum best value after 30 runs achieved by BSSA3 and BSSA4 is not close to the minimum best value obtained by BSSA1 and BSSA2. This magnifies the preeminence of BSSA1 and BSSA2. The same goes for other tests. If we have a closer look at the boxes provided by BSSA1 and BSSA2, it is fair to conclude that BSSA2 is slightly better than BSSA1 in terms of stability and solution quality.

### Wilcoxon rank sum test

The Wilcoxon rank-sum test [39] is a non-parametric hypothesis test that is used to evaluate whether the distributions of populations obtained from two separate sources are with the same medians or not. In this subsection, Wilcoxon rank-sum tests are implemented to assess the differences among the solutions returned by our proposed algorithms. Specifically, Table 3 displays the *p* values we obtained when testing the solution sets given by BSSA1 against those of BSSA2, BSSA2, and BSSA4, respectively. Note that there exists a default significance level *α* = 0.05. In case *p* ≥ *α*, there is not enough statistical evidence to confirm that the difference between the compared populations is significant. Otherwise, it can be concluded that the dissimilarity among the two related sets of values is notable.

**Table 3:** *p*-values returned from the Wilcoxon rank sum test among BSSA1 with other proposed algorithms.

Instance	BSSA2	BSSA3	BSSA4
IDKP1	0.001953	3.81e-07	3.02e-11
IDKP2	0.07483	9.06e-08	3.02e-11
IDKP3	0.03644	3.02e-11	3.02e-11
IDKP4	0.05746	3.02e-11	3.02e-11
IDKP5	0.652	5.08e-03	3.02e-11
IDKP6	0.4035	3.02e-11	3.02e-11
IDKP7	0.7283	3.02e-11	3.02e-11
IDKP8	0.9823	3.02e-11	3.02e-11
IDKP9	0.3112	3.02e-11	3.02e-11
IDKP10	0.1669	3.02e-11	3.02e-11
SDKP1	0.01441	6.70e-11	3.02e-11
SDKP2	0.2707	3.02e-11	3.02e-11
SDKP3	6.20e-04	3.02e-11	3.02e-11
SDKP4	0.4119	3.02e-11	3.02e-11
SDKP5	0.3478	3.02e-11	3.02e-11
SDKP6	0.04207	3.02e-11	3.02e-11
SDKP7	1.07e-07	3.02e-11	3.02e-11
SDKP8	7.74e-06	3.02e-11	3.02e-11
SDKP9	3.57e-06	3.02e-11	3.02e-11
SDKP10	1.70e-08	3.02e-11	3.02e-11
UDKP1	0.00238	4.20e-10	3.02e-11
UDKP2	0.32550	4.08e-11	3.02e-11
UDKP3	0.87660	3.02e-11	3.02e-11
UDKP4	0.46430	3.02e-11	3.02e-11
UDKP5	0.13730	3.02e-11	3.02e-11
UDKP6	0.002755	3.02e-11	3.02e-11
UDKP7	0.08771	3.02e-11	3.02e-11
UDKP8	0.011230	3.02e-11	3.02e-11
UDKP9	1.53e-05	3.02e-11	3.02e-11
UDKP10	9.21e-05	3.02e-11	3.02e-11
WDKP1	0.122400	1.46e-10	3.02e-11
WDKP2	0.004226	3.02e-11	3.02e-11
WDKP3	0.17610	3.02e-11	3.02e-11
WDKP4	0.04207	3.02e-11	3.02e-11
WDKP5	0.9705	3.02e-11	3.02e-11
WDKP6	0.4553	3.02e-11	3.02e-11
WDKP7	0.1023	3.02e-11	3.02e-11
WDKP8	0.001058	3.02e-11	3.02e-11
WDKP9	7.09e-08	3.02e-11	3.02e-11
WDKP10	9.83e-08	3.02e-11	3.02e-11

Based on the statistical results in Table 3, we can conclude that the solutions given by BSSA1 are significantly different from the solutions returned by BSSA3 and BSSA4. The situation between BSSA1 and BSSA2 is more complicated. There are 21 times *p* exceeds the 0.05 threshold, while in the remaining 19 times, *p* is less than 0.05. In another word, in 52.5 percent of the tests, the difference among the solutions given by BSSA1 and BSSA2 is clear, while we can not statistically differentiate them in 47.5 percent cases. In short, Wilcoxon rank sum tests reaffirm what we have observed in previous subsections: BSSA1 and BSSA2 are at the same level and both of them are superior to BSSA3 and BSSA4.

### Friedman test and Nemenyi post-hoc test

This subsection presents the results of the Friedman test [40–42] to provide an additional statistical perspective. Friedman test is a non-parametric test to replace the Repeated Measures ANOVA test [43]. The input parameters for this test are three or more populations, and the test will return a single conclusion after comparing these populations in its way. The null and alternative hypotheses of this test are:

- *H*_0_: The mean values of the populations are similar.
- *H*_*a*_: At least one population mean is different from the mean values of the rest.

Again, the significance level *α* = 0.05 is applied. Suppose the *p*-value returned by the Friedman test is less than or equal to *α*. In that case, the conclusion will be that the null hypothesis is rejected and the alternative hypothesis is confirmed. Otherwise, the null hypothesis will be accepted.

We perform the Friedman test based on the mean fitness values obtained from 30 runs on each instance. Thus, for each in the algorithms BSSA1, BSSA2, BSSA3, BSSA4, we have a population of 40 mean values. These four populations are input parameters for the Friedman test. The returned results of Friedman’s test are as follows:

- Test statistic: 108.66
- *p*-value: 2.1281E-023

The results show that at least one population mean is significantly different from the rest. To clarify which algorithm’s solution population this conclusion is for, we perform the Nemenyi post-hoc test [44]. This test will help answer the question of which population is genuinely distinct. This test returns a table containing the results of pairwise tests. Table 4 shows *p*-values of this test.

**Table 4:** *p*-values from Nemenyi post-hoc test.

	BSSA1	BSSA2	BSSA3	BSSA4
BSSA1	1	0.9	0.001	0.001
BSSA2	0.9	1	0.001	0.001
BSSA3	0.001	0.001	1	0.00299
BSSA4	0.001	0.001	0.00299	1

The results in Table 4 show that, when comparing BSSA1 with BSSA2, the *p*-value is 0.9. When comparing BSSA1 with BSSA3, the p-value is 0.001. The result is the same when comparing BSSA1 with BSSA4. When comparing BSSA2 with BSSA3 and BSSA4, the *p*-values are the same and equal to 0.001. The *p*-value when comparing BSSA3 with BSSA4 is 0.00299. When assessing these values with a significance level of 0.05, it can be seen that BSSA1 and BSSA2 have similar populations of mean total values, and they are significantly different from those of BSSA3 and BSSA4. If we consider the case of BSSA3 and BSSA4, they are also considerably different, although this difference is not as significant as the difference when compared with BSSA1 and BSSA2.

In summary, the Friedman and Nemenyi tests show that the results of BSSA1 and BSSA2 are not significantly different, while they are substantially different from those of BSSA3 and BSSA4.

### Comparison to other algorithms

In this subsection, we compare BSSA1 and BSSA2 with five other algorithms for DKP01. The first is an evolutionary algorithm, FirEGA [8], the second is a swarm intelligence-based one, BPSO8 [14]. Additionally, the best algorithms proposed in [12] and [10], MS1 and MMBO, are also included in the comparison. Note that the two latter algorithms were not tested in inverse correlated data sets, and the related papers did not provide data in some criteria. Anyway, the most important results are available, and they help us in this comparison phase. The results from Improved Harris hawk optimizer (IHHO) [19], a recently published work, are also included in the statistical tables. Although the authors of this algorithm only tested on some representative data sets, we believe that their results help further clarify where our algorithm stands.

Tables 5–8 are used to show the test results. These tables include the statistical calculation results of the total values of the solutions returned after 30 runs of each algorithm. Specifically, column Instance shows the name of the instance tested. Column OPT stores the optimum value, and column Algorithm specifies the algorithm name. At the same time, Best, Average, and Worst present the best, average, and worst values. Meanwhile, Stdev indicates the standard deviation, and Gap reveals the gap between the average and optimum values. Specifically, the Gap value is calculated as specified in the below expression:

$$\begin{array}{c} Gap=\frac{\left|OPT-AVE\right|}{OPT}\times 100\% \end{array}$$ (19)

where AVE stands for the average result.

**Table 5:** Test results with inverse strongly correlated instances.

Instance	OPT	Algorithm	Best	Average	Worst	Stdev	Gap
IDKP1	70106	FirEGA	**70106**	70099.00	70090	7.23	0.01
		BPSO8	69950	69760.97	69521	109.15	0.49
		IHHO	70079	70037.00	-	39.84	-
		BSSA1	**70106**	70098.20	70052	12.27	0.01
		BSSA2	**70106**	**70106.00**	**70106**	**0.00**	**0.00**
IDKP2	118268	FirEGA	118169	117869.00	117625	102.59	0.34
		IHHO	118256	118240.00	-	20.78	-
		BPSO8	118000	117418.00	117040	228.83	0.72
		BSSA1	**118268**	118259.33	118230	16.80	0.01
		BSSA2	**118268**	**118260.00**	**118240**	**5.48**	**0.00**
IDKP3	234804	FirEGA	234497	233997.00	233666	175.42	0.34
		BPSO8	234540	234253.67	233800	192.52	0.23
		IHHO	**234769**	**234760.00**	-	14.43	-
		BSSA1	234750	234739.33	**234730**	**6.91**	**0.03**
		BSSA2	234760	234743.67	**234730**	7.65	**0.03**
IDKP4	282591	FirEGA	282148	280695.00	278881	827.63	0.67
		BPSO8	281560	280953.33	280220	328.28	0.58
		BSSA1	**282590**	282585.33	282570	7.30	**0.00**
		BSSA2	**282590**	**282589.67**	**282580**	**1.83**	**0.00**
IDKP5	335584	FirEGA	335004	333484.00	329621	1173.90	0.63
		BPSO8	334330	333380.67	332480	479.64	0.66
		BSSA1	**335580**	**335580.00**	**335580**	**0.00**	**0.00**
		BSSA2	**335580**	**335580.00**	**335580**	**0.00**	**0.00**
IDKP6	452463	FirEGA	451680	449863.00	446704	1161.52	0.58
		BPSO8	451390	450455.00	448980	563.12	0.44
		BSSA1	**452430**	**452411.00**	452380	12.42	**0.01**
		BSSA2	452420	452410.67	**452390**	**10.81**	**0.01**
IDKP7	489149	FirEGA	488009	485592.00	476385	2294.28	0.73
		BPSO8	485920	484205.67	482400	879.02	1.01
		BSSA1	**489150**	**489130.67**	489100	11.43	**0.00**
		BSSA2	489140	489130.33	**489110**	**11.29**	**0.00**
IDKP8	533841	FirEGA	533035	529984.00	514196	2308.11	0.72
		BPSO8	528450	526356.67	524150	1268.72	1.40
		BSSA1	**533840**	533824.67	**533820**	5.71	**0.00**
		BSSA2	**533840**	**533825.00**	**533820**	**6.82**	**0.00**
IDKP9	528144	FirEGA	526410	523982.00	511651	2216.13	0.79
		BPSO8	522140	518834.67	515480	1726.89	1.76
		BSSA1	**528140**	**528138.33**	528110	5.92	**0.00**
		BSSA2	**528140**	**528138.33**	**528130**	**3.79**	**0.00**
IDKP10	581244	FirEGA	578903	576772.00	568903	1905.18	0.77
		BPSO8	572260	569364.67	566140	1446.41	2.04
		BSSA1	**581240**	**581237.33**	**581230**	**4.50**	**0.00**
		BSSA2	**581240**	581232.00	581130	20.41	**0.00**

**Table 6:** Test results with strongly correlated instances.

Instance	OPT	Algorithm	Best	Average	Worst	Stdev	Gap
SDKP1	94459	FirEGA	93235	93171.00	93070	42.15	1.36
		BPSO8	**94363**	94181.63	93897	113.74	**0.29**
		IHHO	93296	93212.00	-	42.03	
		MS1	94030	93695.00	93376	-	-
		MMBO	93686	93222.00	92371	-	-
		BSSA1	94286	94238.13	94140	36.95	34.42
		BSSA2	94286	**94265.27**	**94224**	**16.37**	34.46
SDKP2	160805	FirEGA	159159	159004.00	158859	61.54	1.12
		BPSO8	**160570**	**160136.00**	**159780**	182.33	**0.42**
		IHHO	159548	159529.50	-	68.59	-
		MS1	159019	158082.00	155574	-	-
		MMBO	159881	159442.00	158433	-	-
		BSSA1	159980	159837.67	159690	70.55	35.15
		BSSA2	159980	159851.33	159720	**58.88**	35.16
SDKP3	238248	FirEGA	235454	235241.00	235043	79.86	1.26
		BPSO8	**237370**	**236778.33**	235960	314.48	**0.62**
		IHHO	235608	235478.00	-	0.00	-
		MS1	236634	236070.00	235765	-	-
		MMBO	236896	236208.00	232114	-	-
		BSSA1	236420	236329.67	236190	**50.82**	0.65
		BSSA2	236510	236379.33	**236210**	56.75	0.67
SDKP4	340027	FirEGA	336353	335963.00	335709	122.41	1.20
		BPSO8	338240	337433.00	336210	495.98	**0.76**
		MS1	337954	337248.00	**336834**	-	-
		MMBO	**338392**	**337522.00**	336733	-	-
		BSSA1	336980	336833.33	336700	65.04	19.19
		BSSA2	336940	336839.00	336690	**50.67**	19.20
SDKP5	463033	FirEGA	452900	447587.00	444255	1974.99	3.34
		BPSO8	459970	458292.00	457130	802.76	**1.02**
		MS1	455491	454026.00	452553	-	-
		MMBO	457678	454344.00	452356	-	-
		BSSA1	**460240**	**460072.67**	**459920**	79.13	37.10
		BSSA2	460150	460051.33	459910	**56.86**	37.09
SDKP6	466097	FirEGA	459254	458893.00	458584	162.94	1.55
		BPSO8	**462650**	**461310.67**	459880	680.64	**1.03**
		MS1	461242	460729.00	460178	-	-
		MMBO	462237	460603.00	457323	-	-
		BSSA1	461020	460871.67	**460780**	**55.53**	1.86
		BSSA2	461030	460831.67	460590	94.03	1.85
SDKP7	620446	FirEGA	599361	592279.00	579673	3949.03	4.54
		BPSO8	614830	613182.67	610700	984.83	**1.17**
		MS1	609852	608712.00	604763	-	-
		MMBO	614167	610971.00	606124	-	-
		BSSA1	**615960**	**615810.33**	**615720**	**63.71**	25.89
		BSSA2	615850	615686.00	615410	90.12	25.87
SDKP8	670697	FirEGA	661276	660104.00	659367	426.06	1.58
		BPSO8	**665230**	663681.67	661500	925.22	**1.05**
		MS1	663804	663103.00	662574	-	-
		MMBO	665183	663766.00	649495	-	-
		BSSA1	664800	**664666.67**	**664540**	**69.30**	24.51
		BSSA2	664710	664527.00	664130	134.94	24.48
SDKP9	739121	FirEGA	729135	727544.00	727064	343.67	1.57
		BPSO8	732910	730950.33	728990	1024.07	**1.11**
		MS1	731439	730654.00	730204	-	-
		MMBO	**734825**	**733517.00**	**732477**	-	-
		BSSA1	731800	731600.33	731440	**79.85**	38.52
		BSSA2	731640	731449.33	731070	148.97	38.49
SDKP10	765317	FirEGA	756205	753394.00	750757	985.46	1.56
		BPSO8	758110	756337.33	753730	1038.86	**1.17**
		MS1	757821	757466.00	757158	-	-
		MMBO	**760814**	**759625.00**	**757750**	-	-
		BSSA1	756250	756100.33	755900	**85.16**	30.08
		BSSA2	756140	755840.67	755340	191.08	30.04

**Table 7:** Test results with uncorrelated instances.

Instance	OPT	Algorithm	Best	Average	Worst	Stdev	Gap
UDKP1	85740	FirEGA	80593	79103.00	77935	690.01	7.74
		BPSO8	**85740**	**85590.03**	**85347**	102.30	**0.17**
		IHHO	79302	78521.00	-	1060.36	-
		MS1	84200	82763.00	81131	-	-
		MMBO	82703	79406.00	76453	-	-
		BSSA1	85186	85036.47	84883	**73.00**	21.30
		BSSA2	85346	85099.07	84983	78.00	21.39
UDKP2	163744	FirEGA	155039	151662.00	149875	1044.95	7.38
		BPSO8	**163560**	**163061.67**	**162260**	314.45	**0.42**
		IHHO	149905	149477.67	-	1630.47	-
		MS1	161133	158503.00	155911	-	-
		MMBO	158465	155976.00	153570	-	-
		BSSA1	161390	160900.33	160550	184.14	36.05
		BSSA2	161140	160914.67	160700	**112.36**	36.06
UDKP3	269393	FirEGA	246698	240886.00	237980	1491.97	10.58
		BPSO8	**268430**	**267008.67**	265560	681.78	**0.89**
		IHHO	237418	234706.00	-	4317.54	-
		MS1	251954	249646.00	244938	-	-
		MMBO	253629	246651.00	242352	-	-
		BSSA1	266810	266517.33	266270	135.92	13.51
		BSSA2	266700	266505.67	**266280**	**100.85**	13.50
UDKP4	347599	FirEGA	321605	317319.00	314486	1426.85	8.71
		BPSO8	**346120**	**343789.00**	341800	924.93	**1.10**
		MS1	332554	320776.00	315150	-	-
		MMBO	333253	329155.00	315914	-	-
		BSSA1	343620	343400.33	**343160**	**136.95**	21.52
		BSSA2	343630	343423.33	343070	138.27	21.53
UDKP5	442644	FirEGA	405409	399620.00	395367	1692.23	9.72
		BPSO8	**437870**	435053.00	431500	1572.94	**1.71**
		MS1	405222	400653.00	395533	-	-
		MMBO	414526	403898.00	395473	-	-
		BSSA1	435470	**435173.00**	**434850**	**128.17**	29.68
		BSSA2	435490	435113.00	434750	167.40	29.66
UDKP6	536578	FirEGA	486556	478726.00	474015	2233.61	10.78
		BPSO8	**530600**	527004.67	523880	1877.81	**1.78**
		MS1	487014	481401.00	476628	-	-
		MMBO	486156	480552.00	474406	-	-
		BSSA1	529070	**528514.00**	**528190**	183.48	16.81
		BSSA2	528740	528375.67	528150	**148.99**	16.78
UDKP7	635860	FirEGA	568119	560948.00	556938	2441.80	11.78
		BPSO8	627350	621875.33	617290	2120.14	**2.20**
		MS1	618146	604287.00	588175	-	-
		MMBO	615617	608351.00	599086	-	-
		BSSA1	**628920**	**628517.33**	**628220**	**146.94**	28.49
		BSSA2	628750	628432.33	627950	177.39	28.47
UDKP8	650206	FirEGA	590137	585286.00	580684	2078.87	9.99
		BPSO8	637900	634388.33	629600	2121.45	**2.43**
		MS1	596452	581196.00	575279	-	-
		MMBO	617036	610379.00	603906	-	-
		BSSA1	**639890**	**639421.00**	**639190**	**154.75**	19.78
		BSSA2	639840	639288.67	638810	225.81	19.75
UDKP9	718532	FirEGA	655172	649636.00	645012	2023.64	9.59
		BPSO8	704350	699156.33	693320	3132.54	**2.70**
		MS1	661984	652572.00	644955	-	-
		MMBO	687790	683032.00	677702	-	-
		BSSA1	**708650**	**708300.00**	**707930**	**147.86**	34.11
		BSSA2	708570	708091.67	707690	201.70	34.07
UDKP10	779460	FirEGA	712270	706575.00	701545	2013.43	9.35
		BPSO8	**767550**	756768.33	749330	3865.48	**2.91**
		MS1	719003	713858.00	706131	-	-
		MMBO	755675	748568.00	739292	-	-
		BSSA1	763570	**763253.67**	**762900**	**193.27**	31.31
		BSSA2	763390	762995.00	762570	217.55	31.27

**Table 8:** Test results with weakly correlated instances.

Instance	OPT	Algorithm	Best	Average	Worst	Stdev	Gap
WDKP1	83098	FirEGA	82803	82693.00	82592	52.04	0.49
		BPSO8	**83090**	**83014.10**	82649	82.14	**0.10**
		IHHO	82782	82733.50	-	98.29	-
		MS1	83074	82947.00	**82800**	-	-
		MMBO	83024	82524.00	80568	-	-
		BSSA1	82943	82852.20	82732	40.48	18.18
		BSSA2	82924	82865.63	82789	**25.44**	18.20
WDKP2	138215	FirEGA	137704	137584.00	137356	63.23	0.46
		BPSO8	138110	137830.00	137270	161.59	**0.28**
		IHHO	137753	137722.00	-	57.28	-
		MS1	**138143**	**137989.00**	**137840**	-	-
		MMBO	138004	137747.00	137275	-	-
		BSSA1	137900	137836.00	137750	31.25	16.55
		BSSA2	137920	137861.67	**137840**	**23.65**	16.57
WDKP3	256616	FirEGA	254120	253657.00	253307	173.01	1.15
		BPSO8	**256360**	255778.67	254850	325.67	**0.33**
		IHHO	254302	254278.00	-	33.23	-
		MS1	248982	248318.00	247714	-	-
		MMBO	255687	254214.00	249695	-	-
		BSSA1	255950	255880.67	255820	36.95	8.98
		BSSA2	255930	**255889.00**	**255840**	**19.71**	8.98
WDKP4	315657	FirEGA	313966	312849.00	311998	484.76	0.89
		BPSO8	**315040**	314340.67	313360	471.89	**0.42**
		MS1	314905	314612.00	314321	-	-
		MMBO	315007	314621.00	313880	-	-
		BSSA1	**315040**	314967.00	**314930**	31.31	11.46
		BSSA2	315030	**314980.67**	**314930**	**24.63**	11.46
WDKP5	428490	FirEGA	426311	424548.00	423058	798.53	0.92
		BPSO8	427390	426349.67	424950	640.64	**0.50**
		MS1	427530	427173.00	426876	-	-
		MMBO	427666	427038.00	425553	-	-
		BSSA1	**427760**	**427662.33**	**427590**	32.77	27.44
		BSSA2	427720	427659.33	427560	**31.62**	27.44
WDKP6	466050	FirEGA	463185	461672.00	457718	1107.57	0.94
		BPSO8	464040	463228.33	461980	487.82	**0.61**
		MS1	464993	464701.00	464383	-	-
		MMBO	**465222**	464299.00	461746	-	-
		BSSA1	464890	464816.00	**464750**	40.22	2.73
		BSSA2	464870	**464819.33**	464740	**32.26**	2.73
WDKP7	547683	FirEGA	544019	541949.00	538126	1224.68	1.05
		BPSO8	545270	544006.67	542140	779.24	**0.67**
		MS1	545607	545241.00	544912	-	-
		MMBO	**546716**	545823.00	544933	-	-
		BSSA1	546500	**546438.00**	**546360**	**37.08**	11.71
		BSSA2	546490	546416.67	546240	53.33	11.71
WDKP8	576959	FirEGA	573427	571559.00	563253	1495.36	0.94
		BPSO8	574290	572652.67	571520	693.20	**0.75**
		MS1	575387	575126.00	574920	-	-
		MMBO	**575850**	575297.00	573694	-	-
		BSSA1	575550	**575474.00**	**575400**	**36.82**	7.80
		BSSA2	575600	575432.67	575320	55.39	7.79
WDKP9	650660	FirEGA	647477	644820.00	630086	2056.06	0.90
		BPSO8	646910	645133.00	642980	923.83	**0.85**
		MS1	649059	648813.00	648611	-	-
		MMBO	**649871**	**649600.00**	**649135**	-	-
		BSSA1	648840	648712.33	648630	**44.00**	22.83
		BSSA2	648700	648620.00	648390	77.99	22.81
WDKP10	678947	FirEGA	675452	673008.00	668239	1441.96	0.88
		BPSO8	675460	673135.33	670990	1171.04	**0.86**
		MS1	677817	677581.00	677401	-	-
		MMBO	**678464**	**678216.00**	**677800**	-	-
		BSSA1	677500	677428.67	677360	**35.60**	16.55
		BSSA2	677440	677357.00	677290	43.64	16.54

Table 5 shows the superiority of our proposed algorithms over other candidate solutions when they are tested with inverse strongly correlated instances. They lead the ranking table in almost all of the cases. The only circumstances when other algorithms raise their voices are the case of IDKP1 when FirEGA has the same best value as our algorithms, and the case of IDKP3 when IHHO has the best results in Best and Average categories. In general, BSSA1 takes the top place 27 times, while BSSA2 has 36 times on this aspect. It is also worth mentioning that there are 14 times when BSSA1 and BSSA2 share the top rank. Generally, our proposed algorithms lead comfortably in the tests using this instance type.

In the case of strongly correlated instances, the situation has changed. Table 6 stores data related to these tests. BPSO8 proves that it adapts very well to this test by leading the ranking table 19 times in total. The results also show that BSSA1 leads 14 times, BSSA2 does the same 7 times, while IHHO, MS1 and FirEGA step aside in every aspect. It is necessary to note that while there are 10 strongly correlated instances, BPSO8 leads on the gap value in all these 10 times. Their average fitness value is closer to the optimum value than that of the opponents. It also means that, with the remaining 9 times claiming the top place, BPSO8 is not superior to BSSA1 and MMBO, whose numbers are 14 and 8, respectively. Another interesting fact is that no top spot is shared among the tested algorithms in this type of instance.

Moreover, BPSO8 seems to be truly better in attaining the best solution, with 5 times at the top of the table, while the remaining 5 times are taken by MMBO and BSSA1 (3 for MMBO and 2 for BSSA1). It is rather equal when we find the best one in terms of the best average result, when BPSO8, MMBO, and BSSA1 reach the first position 3 times each. When searching for the best candidate by comparing the worst outputs of the tested algorithms after 30 runs, our proposed BSSA1 finishes top 4 times while the closest opponents, MMBO and BSSA2, another variant of our algorithm, reach the top spot 2 times. For MS1 and MMBO have no data on Stdev and Gap, if we exclude rankings on these columns, BSSA1 and BPSO8 have similar overall performance in strongly correlated instances, while MMBO finishes third, not so far behind. In terms of standard deviation, our proposed algorithms lead in all 10 instances, which proves that their returned solutions are more consistent than those from the others. In another approach, BPSO8 has the best average ranking in the Best and Gap categories, and BSSA1 comes out on top in the Average, Worst, and Stdev categories.

Uncorrelated data sets are where the values and the costs are not related much, and it is interesting to see how tested algorithms perform in this type of distribution. Table 7 gives data on the performances of the algorithms in solving these instances. It is a rather equal performance when BPSO8 and BSSA1 take the top rank 23 times each. Interestingly, while BPSO8 is unbeatable in all 10 instances when we look at the gap values, its standard deviation performance is not that great. Furthermore, the Stdev values of our proposed BSSA1 and BSSA2 are much lower than those from BPSO8. Hence, since the returned solutions are concentrated close to the expected value, we can conclude that BSSA1 and BSSA2 are more stable than BPSO8, whose solutions are more dispersed. Finally, IHHO, FirEGA, MS1, and MMBO are not really in good form with this type of DKP01 problem with no wins. In terms of average ranking, it is interesting that BPSO8 and BSSA1 lead in the same categories as in the case of strongly correlated instances.

Weakly correlated instances are where MMBO has its voice. Table 8 shows that MMBO gets the first rank in terms of best solutions 5 times. These tests also prove the solid performance of our proposed algorithms. BPSO8 performs best on gap values with 10 wins, while IHHO and FirEGA struggle with every aspect of the tests with zero wins. If we count only the total times claiming the winner spot in the Best, Average, and Worst categories, MMBO has 9 times, BPSO8 has 4 times, BSSA2 has 6 times while BSSA1 is the winner with 10 first places. Our BSSA1 variant dominates most of the categories in terms of average rankings.

Table 9 summarizes the performance of the tested algorithms through their average rankings. Note that because the results of IHHO are available just for 3 instances for each data set type, their average ranking values are calculated by total ranking value divided by 3 for each data set type. Additionally, because the data sets used in the experiments are very different, we provide the statistical results by data set type to see how algorithms perform with each of them. The lower the average rank is, the better the algorithm operates. Our proposed algorithms, BSSA1 and BSSA2, outperform other algorithms in inverse strongly correlated instances. In the case of strongly correlated and uncorrelated instances, although BPSO8 has the best average best ranks, it cannot repeat that performance level in other factors. After analyzing all the factors, it can be seen that BSSA1 achieves the best average rankings in these two data sets. What happens with the weakly correlated data sets is the repetition of what can be seen with inverse strongly correlated instances, where our algorithms have a big difference compared to others in terms of average rankings. In summary, even though the data sets have significant differences, our proposed algorithms still have good adaptability and give higher quality solutions than other algorithms.

**Table 9:** The average ranks of tested algorithms by data set type.

Data set	Algorithm	Average Best Rank	Average AVG Rank	Average Worst Rank	Average Stdev Rank	Average Gap Rank
IDKP	FirEGA	3.1	3.4	3.6	3.8	3.2
	BPSO8	4.2	4.0	3.3	3.6	3.7
	IHHO	2.7	2.7		3.3	
	BSSA1	1.2	1.7	1.7	1.7	1.6
	BSSA2	1.3	1.4	1.1	1.3	1.3
SDKP	FirEGA	6.1	6.2	5.4	3.3	2.2
	BPSO8	1.7	2.5	3.6	4.1	1.0
	IHHO	5.7	5.3		2.3	
	MS1	4.3	4.5	3.6		
	MMBO	2.6	3.3	4.2		
	BSSA1	3.0	2.4	1.9	1.7	3.5
	BSSA2	3.5	2.6	2.3	1.7	3.3
UDKP	FirEGA	5.8	5.9	5.7	3.7	2.0
	BPSO8	1.6	2.2	2.6	3.3	1.0
	IHHO	7.0	7.0		5.0	
	MS1	4.7	4.7	4.7		
	MMBO	4.5	4.4	4.6		
	BSSA1	2.0	1.7	1.5	1.3	3.7
	BSSA2	2.4	2.1	1.9	1.7	3.3
WDKP	FirEGA	6.0	6.1	5.5	3.9	2.0
	BPSO8	3.6	4.3	4.7	3.5	1.0
	IHHO	6.0	5.0		3.3	
	MS1	3.3	3.2	2.9		
	MMBO	2.2	3.6	3.8		
	BSSA1	2.7	2.2	1.8	1.7	3.5
	BSSA2	3.4	2.2	2.1	1.4	3.5

### Computational cost

In this subsection, we will provide an overview of our proposed algorithm’s running time compared to BPSO8. Since PSO is very popular and widely used, we decided to use it as the benchmark algorithm in this test. This comparison allows us to see the computational time of our BSSA2 algorithm compared to a widely recognized optimization algorithm. To perform this assessment, we run the algorithms on uncorrelated instances (UDKP1-UDKP10). Each algorithm will run 30 times on each instance, then the average running time of 30 runs is calculated. Test results show that our algorithm needs more time to finish a run. This could be due to the differences in the operations of the global search algorithm. Anyway, the time required is still acceptable.

Table 10 provides the results of this test.

**Table 10:** Average run time of BSSA2 and BPSO8.

Instance	Average run time (seconds)	
	BSSA2	BPSO8
UDKP1	0.63	0.524
UDKP2	2.879	2.183
UDKP3	6.948	5.009
UDKP4	12.247	9.303
UDKP5	18.656	14.636
UDKP6	26.567	21.14
UDKP7	36.301	29.296
UDKP8	47.815	38.612
UDKP9	59.097	49.688
UDKP10	73.852	61.295

## Conclusions and future work

Discounted {0-1} knapsack problem (DKP01) is not just a theoretical problem but also a principle widely applied in real life. Therefore, finding an effective way to solve this problem will help in real-world business and real-time decision-making systems. Using metaheuristics to solve NP-hard problems, our paper proposes and evaluates a new optimization algorithm with four variants based on the salp swarm algorithm that integrates many new techniques and results in better solution quality. This quality is worth the additional computational cost. The new algorithm is also more stable in producing good solutions than existing ones.

Although the performance of our algorithm is optimistic, some aspects can be further studied in the future. Firstly, in the current approach, while SSA is responsible for covering the search space, the repair operator is responsible for correcting errors that the solutions provided by SSA might make and optimizing it in a predetermined strategy. The problem is that SSA’s exploration capability is somewhat limited, and tweaks are needed to make the global search capacity stronger. Simply put, the current mechanism makes this algorithm very powerful in exploiting a certain direction as well as searching the neighborhoods of the salps in the chain. However, if the algorithm is modified and improved reasonably, later salps can significantly contribute to the exploration process. Improving this property will make the algorithm more powerful and flexible. Next, the repair operator can also be improved. We will be testing various options to make the repair operator work even better, such as defining a new item partitioning scheme. It is also important to note that the sizes of the problem instances in the test data sets are only from 100 to 1,000 dimensions. In the case of more complex data sets, such as 10,000 or 100,000 dimensions or even more, current algorithms for DKP01 will reveal their weakness in terms of computational cost. That’s also an approach we plan to focus on, specifically developing parallel versions of the algorithm that take advantage of the computing power of next-generation CPUs and GPUs and reduce the computational cost.
